# A novel shared decision-making (SDM) tool for anticoagulation management in atrial fibrillation: protocol for a prospective, cluster randomized controlled trial

**DOI:** 10.1186/s13063-023-07667-5

**Published:** 2023-10-02

**Authors:** Mang-Mang Pan, Chi Zhang, Long Shen, Jing-Jing Sha, Hui Shen, Yi-Dan Yan, Jia Wang, Xin Wang, Hou-Wen Lin, Zhi-Chun Gu

**Affiliations:** 1 Department of Pharmacy, Ren Ji Hospital, Shanghai Jiao Tong University School of Medicine, Shanghai, 200127 China; 2 School of Medicine, Tongji University, Shanghai, 200092 China; 3 Department of Cardiology, Ren Ji Hospital, Shanghai Jiao Tong University School of Medicine, Shanghai, 200127 China; 4 Shanghai Pudong New Area, Jinyang Community Health Service Center, Shanghai, 200136 China; 5 Shanghai Pudong New Area, Huamu Community Health Service Center, Shanghai, 201204 China

**Keywords:** Atrial fibrillation, Shared decision-making, Net clinical benefit, Anticoagulation, Warfarin, Non-vitamin K antagonist oral anticoagulants

## Abstract

**Background:**

Atrial fibrillation (AF) is a common arrhythmia that requires anticoagulation therapy to prevent stroke. However, there is still a significant under-/over-treatment in stroke prevention for patients with AF. The adherence and the risk of bleeding associated with oral anticoagulation therapy (OACs) are major concerns. Shared decision-making (SDM) is an approach that involves patients and healthcare providers in making decisions about treatment options. This study aims to assess the effectiveness of a novel SDM tool for anticoagulation management in AF.

**Methods:**

The study will be a prospective, cluster randomized controlled trial involving 440 patients with AF in 8 community health service centers (clusters) in Shanghai, China. The SDM group will receive anticoagulation management through the novel SDM tool, while the control group will receive standard care. The follow-up period will be at least 2 years. The primary outcome will be any bleeding event, while secondary outcomes include the accordance of stroke prophylaxis for AF according to the current guidelines, time in therapeutic range (TTR), the occurrences of major bleeding and thrombosis events, and patient knowledge, adherence, and satisfaction.

**Discussion:**

This study will provide evidence of the effectiveness of shared decision-making in improving the appropriateness of OAC use in Chinese AF patients. The findings may inform the development of guidelines and policies for the management of AF and anticoagulation therapy in China and other countries.

**Trial registration:**

ChiCTR ChiCTR2200062123. Registered on 23 July 2022.

**Supplementary Information:**

The online version contains supplementary material available at 10.1186/s13063-023-07667-5.

## Background

Atrial fibrillation (AF) is a chronic cardiac arrhythmia that is prevalent worldwide, affecting 2 to 4% of the population [1]. In China alone, it affects more than 10 million patients [2]. It is crucial to note that AF substantially increases the risk of stroke by fivefold [3]. Several studies have established that oral anticoagulants (OAC), including warfarin or non-vitamin K antagonist oral anticoagulants (NOACs), are essential for preventing stroke in AF patients [4–7]. However, in clinical practice, suboptimal OAC usage is a common occurrence [8]. Over a third of AF patients who are eligible for OAC treatment receive antiplatelet therapy instead [9]. The Shanghai cross-sectional survey revealed that only 29.5% of AF patients received anticoagulation, and 40.1% were treated with antiplatelet agents for stroke prevention [10]. Additionally, more than a quarter of patients receiving OAC are underdosed or overdosed [11]. Approximately 90% of the study population in Taiwan received a lower dosage of NOACs in 2016 [12]. A Korean national study showed that 40–60% of patients were also provided with lower doses of NOACs [13]. Our epidemiological meta-analysis of 23 studies involving 162,474 AF patients found that the overall prevalence of off-label NOAC doses was 24%, with underdosing accounting for 20% and overdosing for 5% [14]. The inappropriate use of OACs can be attributed to various factors, including clinicians’ lack of understanding of the indications and recommended use of OACs and patients’ excessive concerns about the bleeding risk associated with OACs [15–17]. Patient adherence is also a significant factor affecting anticoagulation quality. After 1 year, medication adherence is at 70% among AF patients, dropping to 50% at 2 years and only 35% after 5 years [18]. In conclusion, inappropriate usage and non-adherence to OAC can result in poor outcomes and high costs for AF patients [18, 19].

Shared decision-making (SDM) is a crucial process that involves both medical professionals and patients in treatment decision-making, leading to improved rationality, reduced decision conflict, and increased patient satisfaction and adherence [1]. This approach is now recommended as a class I recommendation for patient management in clinical guidelines for AF [1]. Although several online tools have been developed to facilitate SDM in AF patients [20–23], most of them use outdated or nonspecific data and lack support for full-process follow-up, making it challenging to achieve optimal individualization of anticoagulation and sustainable management [23]. In response to these challenges, our team developed a SDM tool called I-Anticoagulation, which comprises two functional modules—an anticoagulation decision support system and a full-process patient management system—to enhance the decision-making and patient management of stroke prevention in AF [24]. In the I-Anticoagulation tool, risk estimation for stroke and bleeding is based on Asian patient data, and the corresponding NCB of each optimal anticoagulation strategy are calculated. Clinicians and patients can use this tool to gain a clear understanding of the risks of anticoagulation therapy and engage in a conversation to analyze the advantages and disadvantages of available recommendations and make a shared decision. During the follow-up period, the CHA2DS2-VASc score and the HAS-BLED score are updated with the changes in patient risk factors, and the NCB for each anticoagulant and corresponding recommendations also change dynamically. Accurate recognition of patient risk and precise recommendations for anticoagulation can improve the rationality of anticoagulation therapy, as well as patient adherence and satisfaction. In a pilot study, the use of I-Anticoagulation improved rationality, adherence, and satisfaction in both medical professionals and patients [24]. However, the pilot study had a limited sample size, and only patients using I-Anticoagulation were included in the analyses. Therefore, this study aims to evaluate the effectiveness of the SDM tool in improving the quality, patient adherence, and satisfaction of anticoagulation therapy in AF patients through a prospective, multicenter, cluster randomized controlled trial. In addition to these primary objectives, our research also contains exploratory objectives that aim to broaden our understanding of the SDM tool’s impacts:

- To assess the impact of the SDM tool on patient-clinician provider communication and explore if the tool enables a more effective and interactive dialog around anticoagulation management.
- To evaluate the cost-effectiveness of the SDM tool within the scope of community health service centers. This assessment will look at both direct and indirect medical costs related to anticoagulation management and will aim to discern if the SDM tool can bring about economic benefits by reducing overall cost.

## Methods

### Study design

**Fig. 1:**
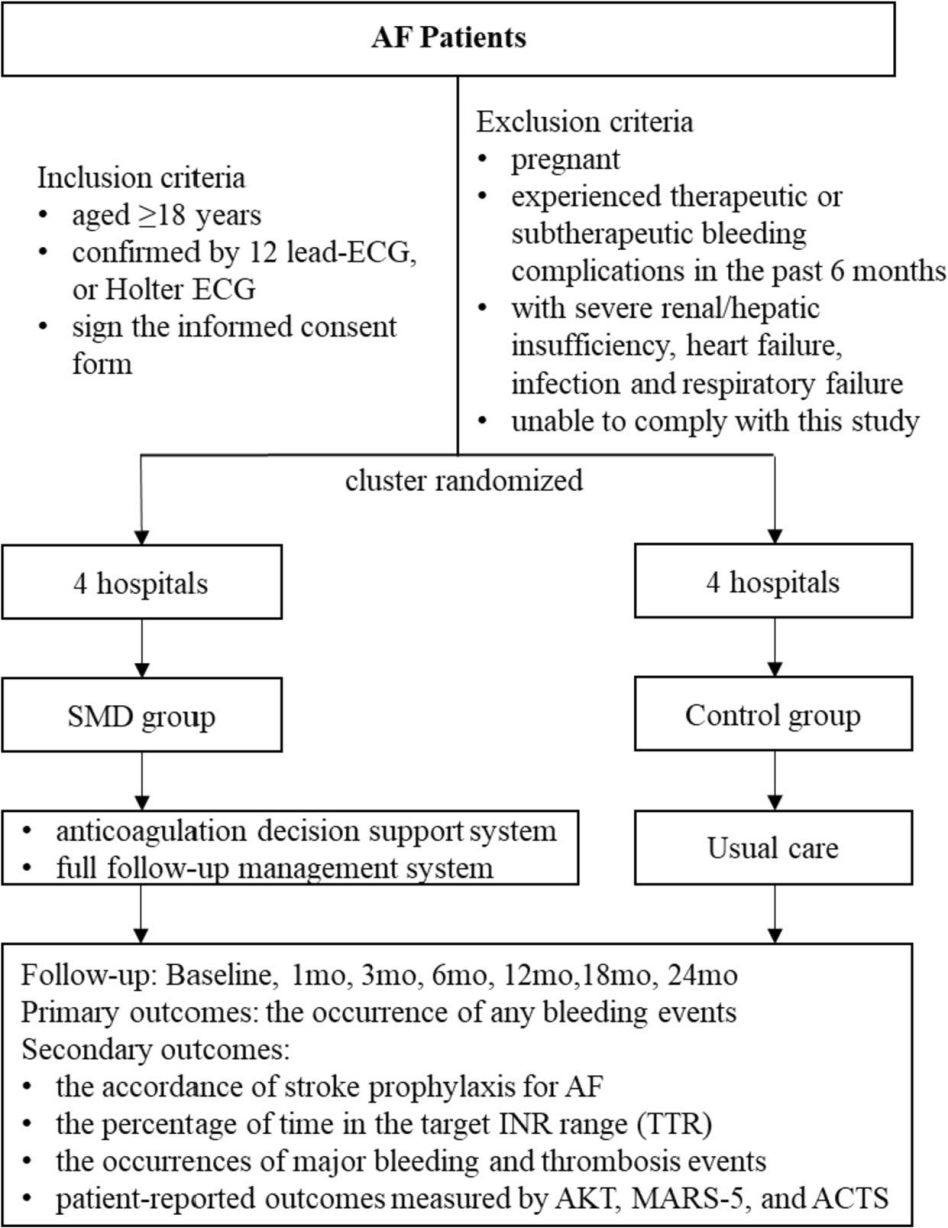
Flow chart of the study. AF, atrial fibrillation; ECG, electrocardiogram; SMD, shared decision-making; TTR, the percentage of time in the target INR range; AKT, Anticoagulation Knowledge Tool; MARS-5, Medication Adherence Reporting Scale; ACTS, Anti-Clot Treatment Scale

This study will be a cluster randomized controlled trial conducted from October 2022 to April 2024 in eight community health service centers in Shanghai, China. The trial will compare the efficacy of using the shared decision-making (SDM) tool, I-Anticoagulation, versus the usual care in treating patients with AF. The clusters will be randomly assigned to either the SDM or control group, as depicted in Fig. 1. Consecutive patients with AF will be enrolled in the study, with those in the SDM group being treated using the I-Anticoagulation tool, while those in the control group receiving standard care. The study was registered on July 23, 2022, in the Chinese Clinical Trial Registry, with the registration number ChiCTR2200062123. This protocol was designed and described according to the standard protocol item—Recommendations for Interventional Trials–Artificial Intelligence (SPIRIT-AI) extension published in 2019 [25] (see Additional file 1).

### Patient and public involvement

Neither the public nor the patients were engaged in the proposal of the research question, the design or implementation of the study, or patient recruitment. The results will be dispersed to the study participants via public reports and academic papers.

### Settings and participants

This study will enroll patients who are 18 years or older and have received a new diagnosis of paroxysmal, persistent, or permanent AF confirmed by electrocardiogram (ECG) or 24-h Holter monitors and are willing to participate in the study and sign the informed consent. Patients who meet any of the following criteria will be excluded: those who are pregnant, have experienced therapeutic or subtherapeutic bleeding complications in the last 6 months, have severe renal insufficiency (creatinine clearance rate, CrCl ≤ 20 ml/min), have severe hepatic insufficiency (Child–Pugh ≥ 10 points), have severe heart failure (cardiac function New York Heart Association, NYHA grade IV and above), have severe infection and respiratory failure, or are unable to comply with the study requirements. Patients can reserve the right to drop out due to any reason or without reason and at any time during the study. If any incident happens to the patients, such as death, disability, or dementia, they could withdraw from the study after applying by their legal representatives and evaluating by researchers. The full eligibility criteria are listed in Table 1.

**Table 1:** Inclusion and exclusion criteria

**Inclusion criteria**
Aged ≥ 18 years
Diagnosed with new-onset, paroxysmal, persistent, or permanent AF confirmed with electrocardiogram (ECG) or 24 h Holter monitors
Patients or guardians agree to the study plan and sign the informed consent form
**Exclusion criteria**
Pregnant patients
Patients who experienced therapeutic or subtherapeutic bleeding complications in the past 6 months
Patients with severe renal insufficiency (CrCl ≤ 20 ml/min)
Patients with severe hepatic insufficiency (Child–Pugh ≥ 10)
Patients with severe heart failure (NYHA grade IV and above)
Patients with severe infection and respiratory failure

*AF* atrial fibrillation, *CrCl* creatinine clearance rate, *NYHA* New York Heart Association

### Recruitment

In order to ensure a sufficient number of participants, the recruitment methods will involve a combination of some approaches. Firstly, electronic medical records will be used to identify the potential participants who meet the inclusion criteria. Secondly, these potential patients will be reached out through mailings and phone calls. Additionally, we will establish a collaboration with physicians across various departments in community health centers involved in the research, to refer patients who meet the criteria to our study. To enhance recruitment, advertisements on online platforms and social media, such as WeChat Official accounts, will be used to raise awareness about the study and encourage participation.

Each center will aim to recruit approximately the same number of participants per month to ensure a balanced distribution of participants across the study duration. Based on this, the estimated recruitment per center per month will be approximately 3 participants (440 participants/18 months/8 centers).

After the screening, the participants who meet the eligibility criteria will be recruited. To ensure a comprehensive and efficient recruitment process, a trained doctor in the department of cardiology from each community health service center involved in the study will collaborate with a clinical pharmacist to facilitate and monitor the recruitment process. In addition, each center will have a dedicated research assistant who will serve as the project’s focal person throughout the study duration. The contact information of recruited participants will be collected and securely stored. The potential participants will be provided with detailed information about the study both in written and verbal formats. They will also be given consent forms, which will clearly state their rights to withdraw from the study at any point. Once the participants have been fully informed and have provided their consent, they will be enrolled in the study and assigned unique code numbers by the principal investigator (PI).

### Randomization and blinding

To avoid any risk of contamination between the SDM and control groups, cluster randomization will be implemented in this trial. The intervention provided to patients with AF will be grouped by clinicians in a single community health service center. Computer-generated randomization will be used to allocate the intervention and control groups in a 1:1 ratio following AF confirmation. The allocation results of each community health center will be told to the administrators by telephone. Each administrator only knows the result of his or her own site. The researchers, participants, and data analysts will not be blinded, but the participants will not be allowed to know the outcomes of this study.

### Interventions

#### Introduction

Community health service centers that are randomized to the SDM group will utilize the SDM tool to manage AF patients. The medical specialists involved in this study will have access to the SDM tool. The anticoagulation regimens for AF patients will be determined through communication between the clinicians and patients based on the suggestions and information provided by the SDM tool. This includes anticoagulation strategies that have a corresponding net clinical benefit (NCB), cost of each anticoagulant, blood examination of warfarin, and other relevant considerations. Clinicians will prescribe dosage regimens and order appropriate laboratory monitoring based on the recommendations of the SDM tool. Furthermore, this SDM tool can also assist clinicians with anticoagulation regimes in AF patients with specific circumstances, such as adjuvant bridging therapy, periprocedural anticoagulation recommendations, and drug regimen adjustments for end-organ function, weight, and concomitant interactions. With the help of the SDM tool, pharmacists can provide comprehensive patient education, including indications for the therapy, medication intake, medication interactions, laboratory monitoring, activity, diet, side effects, pregnancy, procedures, safety precautions, and self-care. The SDM tool will also enable the tracking of bleeding and other adverse events relevant to anticoagulation, as well as assessing patients’ knowledge, adherence, and satisfaction with anticoagulation therapy. The tool will automatically schedule the follow-up scheme, and the anticoagulation therapy will be reassessed based on the follow-up data of the patients. The records and information of the patients will be obtained from the SDM tool cloud platform. In contrast, the control group will receive the usual care based on the clinicians’ usual approach.

#### Introduction of the anticoagulation SDM tool (I-Anticoagulation)

In a previous study, a novel anticoagulation shared decision-making (SDM) tool was developed with the input of healthcare professionals including physicians, pharmacists, and nurses [24]. To facilitate optimal anticoagulation management, a WeChat Mini application-based tool called I-Anticoagulation was created, which offers a flexible and efficient communication platform for healthcare professionals and AF patients. The tool is composed of two modules: an anticoagulation decision support system and a full-process patient management system.

The anticoagulation decision support system requires the input and recording of baseline information of patients with AF, such as age, weight, gender, smoking, alcohol consumption, diagnosis, complications, comedications, history of stroke and bleeding, blood pressure, liver and kidney function, and heart function [24]. Liver function will be assessed using the Child–Pugh score, and renal function will be calculated using the Cockcroft-Gault formula based on creatinine clearance. The tool can automatically calculate CHA2DS2-VASc and HAS-BLED scores to evaluate the patient’s stroke and bleeding risks, respectively, based on incidence data for Asian AF patients [26–28]. The anticoagulation strategies with NCB values will also be presented on I-Anticoagulation.

Patients on anticoagulant therapy can be enrolled in the full follow-up management system, with patient information updated at each follow-up visit, including adjusted dose, INR value (if warfarin was used), the reason for unstable INR, thromboembolic/bleeding events during anticoagulation, and other changing characteristics. The risks of stroke and bleeding will be dynamically reassessed according to the changes in patient characteristics, and the NCB for each anticoagulant drug will also be recalculated, with dynamic recommendations for appropriate anticoagulation regimens.

In addition, the SDM tool includes a series of questionnaires, such as the Anticoagulation Knowledge Tool (AKT) [29], Medication Adherence Reporting Scale (MARS-5) [30], and Anti-Clot Treatment Scale (ACTS) [31], to evaluate medication knowledge, adherence, and satisfaction of AF patients receiving anticoagulation.

Clinicians can search for recommended anticoagulation strategies based on the latest guidelines or consensus for patients with AF affected by renal/liver impairment, coronary artery disease (CAD), perioperative period, or switching to a different type of anticoagulant.

### Follow-up

In this study, each patient enrolled will be monitored for at least 2 years, regardless of whether they meet the study’s endpoint or not. For both the SDM group and the control group, the same set of questionnaires will be used for consistency in recording follow-up data. The enrolled patients will undergo follow-ups on the 1st, 3rd, 6th, 12th, 18th, and 24th months through outpatient clinic visits. The follow-up plan will be automatically arranged using the SDM tool. Patient anticoagulation therapy will be re-evaluated based on follow-up data, and information on patients’ records and past data will be retrieved from the SDM tool’s cloud platform. At each follow-up time point, patients in the SDM group will complete the questionnaires within the tool, while patients in the control group will complete the questionnaires within the clinical case report form (CRF). A designated medical staff member will send text reminders to patients to attend follow-ups. Patients in the control group who cannot attend scheduled follow-ups at the community health service centers can be followed up by telephone.

### Outcomes

The outcomes will be collected at each follow-up time point (1st, 3rd, 6th, 12th, 18th, and 24th months).

#### Primary outcomes

While thrombotic events are a critical consequence of AF, the incidence rate of these events in patients who are adequately anticoagulated is generally lower than the incidence of bleeding events. Consequently, the study’s primary outcomes comprise any bleeding events, both major and minor. Major bleeding events [32] include fatal hemorrhage, life-threatening bleeding, and severe bleeding requiring treatment or assessment. Severe bleeding can manifest as gastric intestinal hemorrhage, joint hematoma, retroperitoneal hemorrhage, fundus hemorrhage, urine, hemoptysis, or require blood transfusion of at least two units. In contrast, minor bleeding events, such as mild nasal hemorrhage, endoscopic hematuria, skin stasis, or mild hemorrhoids, do not lead to serious consequences [33].

#### Secondary outcomes

**Fig. 2:**
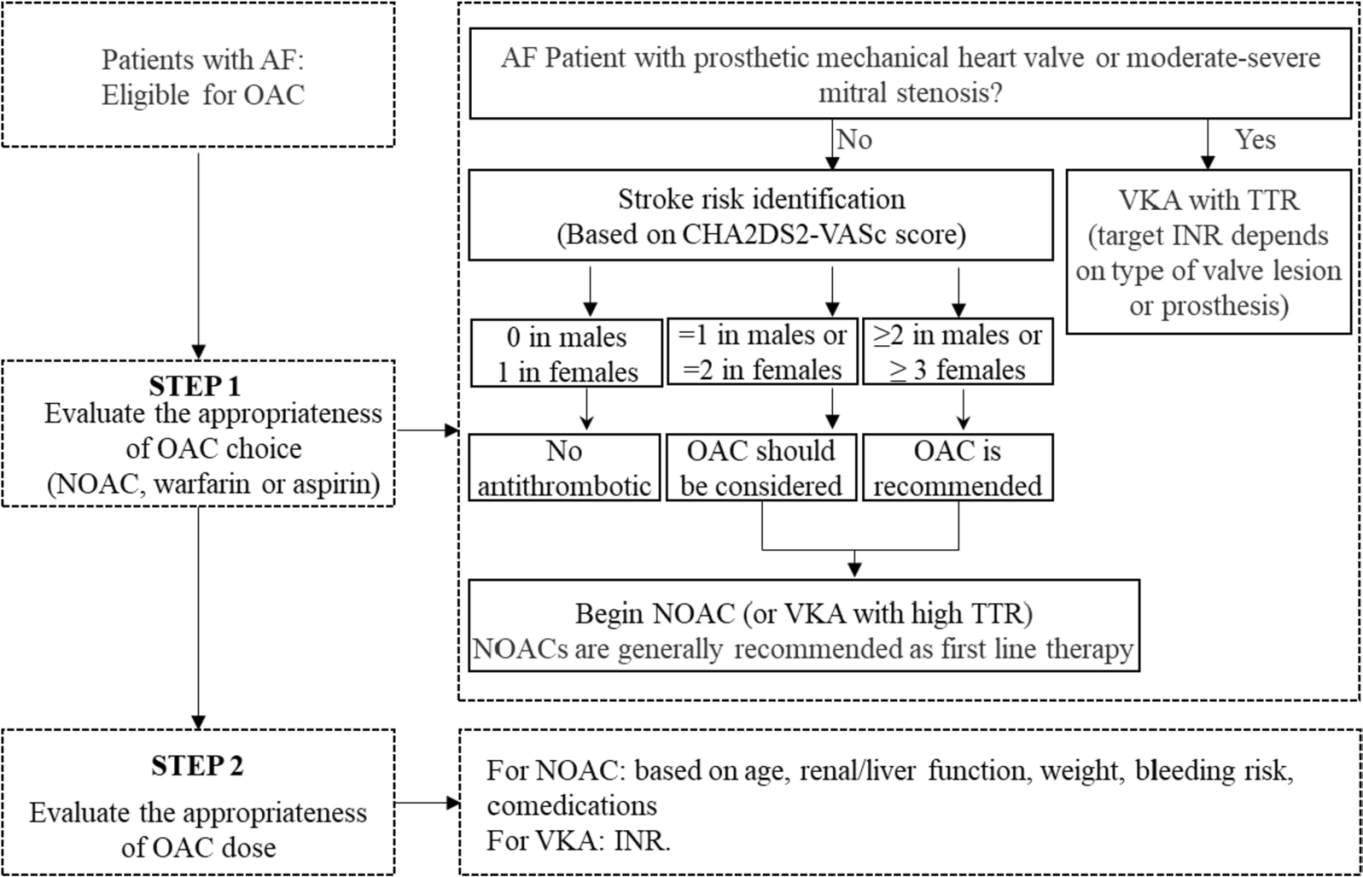
Evaluation flow chart. AF, atrial fibrillation; OAC, oral anticoagulant; NOACs, non-vitamin K antagonist oral anticoagulants; VKA, vitamin K antagonist oral anticoagulant; TTR, the percentage of time in the target INR range

The study’s secondary outcomes include the accordance of stroke prophylaxis for AF according to the current guidelines, the percentage of time in the target INR range for patients using warfarin, the occurrences of major bleeding and thrombosis events, and the OAC knowledge, adherence, and satisfaction of AF patients receiving anticoagulation therapy. The study will evaluate stroke prophylaxis for AF using a flow chart (Fig. 2) and guidelines for dabigatran, rivaroxaban, and edoxaban (Table 2). The anticoagulation quality of warfarin will be assessed using the TTR, and the therapeutic range of INR for patients with AF is set between 1.8 and 3.0 [34]. The study considers TTR levels of 65% or higher as good anticoagulation quality. Thrombosis events, including ischemic stroke, systemic embolism, and transient ischemic attacks, will be confirmed by imaging methods such as ultrasound, CT, or MRI. To evaluate patients’ knowledge, adherence, and satisfaction with anticoagulation therapy, the study will use AKT, MARS-5, and ACTS. The AKT comprises 28 items assessing the general anticoagulation knowledge for all available OAC and a specific section for VKA therapy. The MARS-5 assesses common patterns of nonadherent behavior, and the ACTS evaluates the burdens and benefits of anticoagulation treatment and its overall impact (see Additional file 2).

**Table 2:** The dose-adjustment criteria for the NOACs according to the Chinese labeling

Drugs	Dabigatran	Rivaroxaban	Edoxaban
Standard dosing	150 mg bid	20 mg qd	60 mg qd
Age ≥ 75 years	110 mg bid	15 mg qd	Not mentioned
Weight < 60 kg	Not mentioned	15 mg qd	30 mg qd
Renal impairment dosing	CrCl 30 ~ 50 ml/min: 150 mg bid/110 mg bid (high risk of bleeding); CrCl < 30 ml/min: avoid; dialysis: avoid	CrCl 15 ~ 50 ml/min: 15 mg qd; CrCl < 15 ml/min: avoid; dialysis: avoid	CrCl: 15 ~ 50 ml/min: 30 mg qd; dialysis: avoid
Hepatic impairment	Child–Pugh C: avoid	Child–Pugh B: avoid; Child–Pugh C: avoid	Child–Pugh C: avoid
Drug-drug interaction	110 mg bid: verapamil: avoid with cyclosporin, ketoconazole, itraconazole, dronedarone	Not recommend: fluconazole, itraconazole, ketoconazole, voriconazole, HIV protease inhibitors	30 mg qd: cyclosporin, dronedarone, erythromycin, ketoconazole

*CrCl* creatinine clearance

### Data collection and management

All pertinent information of clinical patients in the group will be documented after enrollment, which includes demographic details, diagnosis, drug usage, test outcomes, and follow-up results. All data will be collected in accordance with standard procedures outlined in Table 3. It is important to note that this trial does not involve collecting biological specimens for storage. The medical information of each patient enlisted in the SDM tool, which is a WeChat mini-program-based model, will be saved in a monitoring database. Professionals will ensure that patients’ personal information is secure and that all files remain confidential. If there are any bugs or data leakage, all people will stop using the SDM until the problem is solved and the ethics committee approves resumption. The users can contact the researchers at any time if they encounter any difficulties, and a response will be given within 24 h. For severe adverse events, researchers will report them to the ethics committee within 24 h. If it is attributed to the SDM tool, the ethics committee and the PI can terminate prematurely and conduct interim analyses. The final trial dataset will be made available to the PIs, research team members, and authorized personnel who are directly involved in the study. These individuals will be granted access to the dataset for the purpose of data analysis, interpretation, and publication of the trial results. Researchers or organizations interested in obtaining specific data related to our trial can make a formal request, and we will strive to provide the requested information promptly while adhering to any applicable data protection laws and regulations. Access to the SDM tool intervention and its corresponding code can be made available upon request to the PI or corresponding author of this study. However, restrictions may apply to protect intellectual property or preserve confidentiality. These limitations will be disclosed transparently during the access request process.

**Table 3:** Schedule of study activities

Time points	Day of admission (D0)	Follow-up (months)					
		1	3	6	12	18	24
**Enrollment**							
Informed consent	×						
Inclusion/exclusion criteria	×						
Demographics^a^	×						
Past medical history^b^	×						
Comedications^c^	×	×	×	×	×	×	×
**Assessment**							
Liver function^d^	×	×	×	×	×	×	×
Kidney function^e^	×	×	×	×	×	×	×
Heart function^f^	×	×	×	×	×	×	×
Stroke risk^g^	×	×	×	×	×	×	×
Bleeding risk^h^	×	×	×	×	×	×	×
Coagulation function	×	×	×	×	×	×	×
Patient-reported outcomes							
AKT^i^	×	×	×	×	×	×	×
MARS-5^j^	×	×	×	×	×	×	×
ACTS^k^	×	×	×	×	×	×	×

^a^Age, height, weight, gender, ethnicity, date of birth, history of smoking, and alcohol

^b^Hypertension, coronary heart disease, diabetes, and history of stroke

^c^Antiplatelet agent and interacting combination medications with NOACs, such as antiarrhythmic therapy, itraconazole, ketoconazole, and ritonavir, etc

^d^Child-Pugh score

^e^CrCl, calculated using Cockroft-Gault formula

^f^LVEF%

^g^CHA2DS2-VASc score is a rating of risk for stroke in patients with AF, items of 1 point each for congestive heart failure, hypertension, diabetes mellitus, vascular disease, age 65–74 years, and sex category [female] and 2 points each for a history of a stroke, TIA, or age ≥ 75 years

^h^HASBLED score is a rating of risk for bleeding in patients with AF, 1 point each for hypertension, abnormal renal/liver function, stroke, bleeding history or predisposition, labile INR, 65 years or older, drugs/alcohol concomitantly

^i^Anticoagulation Knowledge Tool

^j^Medication Adherence Reporting Scale

^k^Anti-Clot Treatment Scale

### Sample size

This multicenter, cluster-randomized clinical trial aims to evaluate the occurrence of bleeding events as the primary outcome. The anticoagulation bleeding rate of patients with AF in our hospital was previously found to be 20% [24], and an effective intervention reduces the incidence of bleeding by 15% [35]. We set the power at 80% with a bilateral two-tailed significance of 5%. To achieve a sample size of 88 cases in both the intervention and control groups, with a 20% loss to follow-up rate, we estimated a minimum of 110 cases in each group. However, taking the clustering into account, we increased the sample size to account for the design effect to achieve the required statistical power under cluster randomization. Therefore, using the bleeding rate as the calculating target and a design effect of 2, we estimated a sample size of 440, which was calculated using the PASS software version 15.

### Statistical analyses

In accordance with standard practice, continuous variables will be presented as means ± standard deviation, while categorical variables will be expressed as proportions. The Kolmogorov–Smirnov test will be utilized to determine the normality of the data. Student’s *t*-test or Mann–Whitney’s *U*-test will be applied to compare two groups, depending on the distribution of the data. For categorical data, comparisons between the groups will be performed using the chi-square or Fisher’s exact tests. Time in therapeutic range (TTR) will be evaluated for patients on warfarin. Multiple imputations will be used to fill in missing values, and both per-protocol (PP) analysis and intention-to-treat (ITT) analysis will be conducted. Subgroup analyses will be performed based on patients’ age groups, treating hospitals, liver or kidney function, the type of anticoagulants used, etc. Statistical significance will be assumed for *p* values less than 0.05. All statistical analyses will be conducted with the IBM SPSS version 22.0 software.

### Confidentiality

All medical data and personal information of the participants will be kept confidential in accordance with the international guidelines and regulations. In addition, the development and use of the I-Anticoagulation tool, which includes the collection and processing of patient information, was based on the national standard GB/T 35273–2020 “Information security technology-Personal information security specification” of China [24]. Thus, all participant information will be treated with the utmost discretion and protection throughout this study. Only designated members of the research team will have access to participant data, and access will be limited to the information necessary to perform their specific duties. Participant identification codes will be used instead of participant names to ensure anonymity and confidentiality. Data sharing and confidentiality agreements will be developed to protect the confidentiality of research data, analytical methods, and results. Participants’ medical data and personal information will be encrypted, password-protected, or stored offline as needed to ensure data security. All documents related to the study, including informed consent forms and participant medical records, will be securely stored in designated locations to ensure confidentiality.

### Oversight and monitoring

#### Composition of the coordinating center and trial steering committee

This study is coordinated by CZ and MMP. The research will be supervised by researchers ZCG and HWL. The establishment of an additional steering committee was not considered for this study. All research personnel will participate in quarterly meetings to discuss research progress and potential unforeseen events. There is no stakeholder and public Involvement Group involved in this study.

#### Composition of the Data Monitoring Committee, its role, and reporting structure

This study has no composition of Data Monitoring Committee (DMC) as this is a low-risk intervention, and other data will be reserved in the information security department as parts of patients’ medical records.

#### Monitoring

The clinical research unit (CRU) of Renji Hospital provides study management, guidance, and supervision for clinical studies, which includes regular monitoring protocol adherence, ensuring informed consent standards, reporting serious adverse events (SAEs), verifying data traceability, and tracking project progress. The CRU’s supervision will ensure our study’s integrity, reliability, and ethical conduct. Throughout the study period, the research team, under the guidance and oversight of the CRU, will regularly inspect the completeness of subjects’ records, accuracy of data input, and patients’ adherence to the study protocol, as well as conducting quarterly quality control meetings to monitor the progression of the group and ensure that all activities are carried out in compliance with the program’s rules.

## Discussion

A prospective, cluster-randomized trial will be conducted to evaluate the effectiveness of a novel SDM tool in reducing bleeding events and improving guideline compliance for stroke prophylaxis in AF patients. Additionally, the trial aims to evaluate the impact of the SDM tool on anticoagulation quality, patient adherence, and satisfaction.

In the current healthcare landscape, pharmacists have become a vital link in the chain of patient care, as they are able to provide patients with critical support when it comes to managing their chronic illnesses and medications, especially when it comes to anticoagulation decisions [36, 37]. Studies have shown that when pharmacists are involved in shared decision-making, patients benefit from better medication appropriateness and adherence [38, 39]. To further explore the potential benefits of pharmacist involvement, our research will incorporate pharmacists as part of the patient care team in a combined pharmacy clinic. Specifically, we will investigate the role of pharmacists in ensuring that anticoagulant prescriptions for the AF management are appropriate and in line with guidelines.

Although clinicians show a wealth of interest and willingness in participating in research, their heavy clinical workload hurdles the progress of clinical studies, especially when they need to manually enter basic patient data into management tools. It will be very convenient and useful to seamlessly integrate data from hospital information systems (HIS) into mobile healthcare tools [40]. By integrating data, mobile healthcare tools can directly capture patient characteristics without relying on the manual input of healthcare workers. Such integration enhances the accuracy and efficiency of health management [40, 41]. Nevertheless, there are no established standards for integrating HIS and mobile healthcare tool data in this study. In addition, ensuring the online safety of patient information is a significant concern, making the process of seamless data integration complex [42]. This process necessitates permissions from various departments such as the Department of Information, the Department of Medicine, and the Ethics Committee. Therefore, more efforts should be made to establish data exchange standards between HIS and mobile healthcare tools, which will accelerate the development of mobile healthcare applications [41].

In the process of this study, standardizing the use of SDM tool across diverse patient populations with varying levels of health literacy and understanding will be a challenge. Accordingly, it is essential for researchers to effectively communicate and engage with patients from different backgrounds and varying levels of health literacy. A shared decision-making tool developed by the Mayo Clinic allowed researchers to train participating clinicians at each research center in interactive demonstrations, providing video clips and demonstrating how to use the dialog tool to communicate with patients [22]. The mAFA trial program delivered the training on mAF App use for the researchers before the study [43]. In this study, the stroke and bleeding risks associated with AF will be visualized, as well as the benefits of anticoagulant use to facilitate effective communication between physicians and patients, making it easier for patients to fully understand. Additionally, to ensure all groups can effectively utilize the tool, a comprehensive training program, including principles and benefits of SDM, strategies for effective communication, and techniques for adapting the use of the SDM tool based on individual patient needs, will also be conducted to overcome operational barriers and ensure consistent and accurate use of the SDM tool across all participating groups.

### Strengths and limitations

This study has significant advantages, as it is a large-scale multicenter cluster randomized controlled trial involving a substantial number of participants. However, there are some limitations to this research. Firstly, the 24-month period may not be long enough to assess the effect on clinical outcomes such as thromboembolic events. Therefore, future studies should consider longer follow-up periods to evaluate the effects of the intervention over an extended period. Secondly, it is possible that those who agreed to participate in the research might be more interested in their own health, leading to a higher adherence to medication therapy than the general population with AF. This could potentially affect the generalizability of the study findings.

## Trial status

This publication is based on version 3.0 of the trial protocol dated June 16, 2022. Patient enrollment started in October 2022. We anticipate enrollment will be completed by April 2024.

### Supplementary Information

**Additional file 1. **SPIRIT-Al checklist.

**Additional file 2. **Questionnaire.

**Additional file 3. **Copy of the original funding documentation.

**Additional file 4. **Ethical approval document.

## Data Availability

The datasets analyzed during the current study and statistical code are available from the corresponding author upon reasonable request.

## Abbreviations

SMD: Shared decision-making
AF: Atrial fibrillation
OAC: Oral anticoagulant
NOACs: Non-vitamin K antagonist oral anticoagulants
VKA: Vitamin K antagonist oral anticoagulant
TTR: The percentage of time in the target INR range
ECG: Electrocardiogram
NCB: Net clinical benefit
CrCl: Creatinine clearance rate
PI: Principal investigator
AKT: Anticoagulation Knowledge Tool
MARS-5: Medication Adherence Reporting Scale
ACTS: Anti-Clot Treatment Scale
CAD: Coronary artery disease
CRF: Case report form
PP: Per-protocol
ITT: Intention-to-treat
DMC: Data Monitoring Committee
CRU: Clinical research unit
