# Supplementary material for: A novel shared decision-making (SDM) tool for anticoagulation management in atrial fibrillation: protocol for a prospective, cluster randomized controlled trial
Source: Trials. 2023 Oct 2;24:623. doi: 10.1186/s13063-023-07667-5 (PMC10544439; doi:10.1186/s13063-023-07667-5)
Supplement: Supplementary file 2 — Additional file 2. Questionnaire. [file 13063_2023_7667_MOESM2_ESM.pdf]

Supplementary Table 1 Anticoagulant Satisfaction Questionnaire

	Not at All	A Little	Moderately	Quite a Bit	Extremely
1. How much does the possibility of bleeding as a result of anticlot treatment limit you from taking part in vigorous physical activities (e.g., exercise, sports, dancing)?	1	2	3	4	5
2. How much does the possibility of bleeding as a result of anticlot treatment limit you from taking part in your usual activities (e.g., work, shopping, housework)?	1	2	3	4	5
3. How bothered are you by the possibility of bruising as a result of anticlot treatment?	1	2	3	4	5
4. How bothered are you by having to avoid other medicines (e.g., aspirin) as a result of anticlot treatment?	1	2	3	4	5
5. How much does anticlot treatment limit your diet (e.g., food or drink, including alcohol)?	1	2	3	4	5
6. How much of a hassle (inconvenience) are the daily aspects of anticlot treatment (eg, remembering to take your medicine at a certain time, taking the correct dose of your medicine)?	1	2	3	4	5
7. How much of a hassle (inconvenience) are the occasional aspects of anticlot treatment (eg, the need for blood tests, going to or contacting the clinic/doctor)?	1	2	3	4	5
8. How difficult is it to follow your anticlot treatment?	1	2	3	4	5
9. How much do you worry about your anticlot treatment?	1	2	3	4	5
10. How much of a burden is your anticlot treatment?	1	2	3	4	5
**11. Overall, how much of a negative impact has your anticlot treatment had on your life?**	1	2	3	4	5
	Not at All	A Little	Moderately	Quite a Bit	Extremely

12. How confident are you that your anticlot treatment will protect you from the stroke?	1	2	3	4	5
13. How safe do you feel because of your anticlot treatment?	1	2	3	4	5
14. How satisfied are you with your anticlot treatment?	1	2	3	4	5
**15. Overall, how much of a positive impact has your anticlot treatment had on your life?**	1	2	3	4	5
**Total (scores)**					

1, Not at all" was calculated as 1 point. "2, A little" was calculated as 2 points, "3, Moderately" was calculated as 3 points. "4, Quite a bit" was calculated as 4 points. "5, extremely" was calculated as 5 points.

Supplementary Table 2 Medication Adherence Report Scale

Item	Always	Often	Sometimes	Rarely	Never
"I forget to take them"	1	2	3	4	5
"I alter the dose"	1	2	3	4	5
"I stop taking them for a while"	1	2	3	4	5
"I decide to miss out a dose"	1	2	3	4	5
"I take less than instructed"	1	2	3	4	5

Non-adherence was defined as reporting any non-adherence on the MARS (total score <25 or at least one MARS-item with score <5).

Supplementary Table 3 Anticoagulation Knowledge Tool (AKT)

No.	No General questions
1	What is the name of your anticoagulant medicine?
2	Why has your doctor prescribed you this medicine?
3	How does this medicine work in your body?
4	How many times a day do you need to take this medicine?
5	For how long do you need to take this medicine (for example, 3 months, and 6 months, life-long)?
6	Why is it important to take this medicine exactly as your doctor has told you?
7	Is it acceptable to take this medicine at different times as long as you take it on the required days?
8	Is it acceptable to double the next dose of this medicine if you miss a dose?
9	Is it possible that skipping one dose of this medicine could worsen your condition?
10	Is it appropriate to stop taking this medicine once you feel better?
11	Is it safe to take anti-inflammatory medicines like ibuprofen (Nurofen1 or Advil1) while you are taking this medicine?
12	Is it safe to take vitamin supplements and herbal medicines with this medicine without consulting your doctor?
13	Is there any benefit in taking more of this medicine than your doctor has told you to take?
14	Will drinking too much alcohol increase the risk of side effects with this medicine?
15	Is it necessary to inform a surgeon, dentist or other health professional that you are taking this medicine before undergoing surgery or a procedure?
16	Is it important that all the health care practitioners you see know that you are taking this medicine?
17	What is the most important side effect of this medicine?
18	Three signs of side effects that you should watch out for while taking this medicine are:

19	Three things you can do to reduce your risk of side effects are:
20	What is the best step to take if you accidentally take too much of this medicine?
**Question specific to people taking warfarin**	
1	What is your target INR range?
2	What was your last INR reading?
3	Are routine INR tests necessary to know how well this medicine is working?
4	Is an INR value above your target range good for your general wellbeing?
5	Is it possible for INR values below your target range to be bad for your health?
6a	Is it possible for your diet to affect your warfarin therapy?
6b	If you answered 'Yes' above, list Three foods that can affect your anticoagulant therapy.
7	List one vitamin that can significantly affect your anticoagulant therapy.

Scoring was done use a dichotomous scale, with a score of '1' or '0' for each correct answer or wrong answer, respectively. A maximum score of '1' was allocated to each correct answer for all of the questions with the exception of item '6', '18' and '19' in section 'A' and item '6b' in section 'B'. A maximum score of '2' was obtainable for item '6' in section 'A'- ('Why is it important to take this medicine exactly as your doctor has told you?') - 1 mark each was allotted for answers related to the prevention of thromboembolism and answers related to minimising the risk of bleeding. For items '18' and '19'- ('three signs of side effects you should watch out for' and 'three things you can do to reduce your risk of side effect', respectively) - 1 mark each was allotted for each correct sign of side effects to look out for and each correct approach to reduce the risk of bleeding. Lastly, for item '6b' in section 'B' ('list three foods that can affect your anticoagulant therapy') - 1 mark each was allotted for three correct food substances mentioned. A maximum total score of '25' was obtainable for patients taking the DOACs required to answer only section 'A' of the questionnaire, while a maximum total score of '35' was obtainable for patients taking the VKAs (warfarin) required to answer both sections of the questionnaire. Final scores were presented as a percentage of correct answers for all the participants in the study.
