# Supplementary material for: A novel shared decision-making (SDM) tool for anticoagulation management in atrial fibrillation: protocol for a prospective, cluster randomized controlled trial
Source: Trials. 2023 Oct 2;24:623. doi: 10.1186/s13063-023-07667-5 (PMC10544439; doi:10.1186/s13063-023-07667-5)
Supplement: Supplementary file 3 — Additional file 3. Copy of the original funding documentation. [file 13063_2023_7667_MOESM3_ESM.pdf]

密级：

上海申康医院发展中心

第二轮《促进市级医院临床技能与临床创新三年行动计划》研究型医师创新转化能力培训项目

项目任务书

项目名称：	基于“爱抗凝”小程序的抗凝决策与管理模式在房颤患者中的应用：一项前瞻性、多中心、整群随机对照研究
项目编号：	SHDC2022CRS035
承担单位（盖章）：	上海交通大学医学院附属仁济医院
项目负责人：	张 弛
执行期限：	2022 年 7 月 1 日至 2024 年 9 月 30 日

上海申康医院发展中心

20 年 月 日

Shanghai Shenkang Hospital Development Center

The Second Round of "Three-Year Plan for Promoting Clinical Skills and Innovation in Municipal Hospitals of Shanghai Shenkang Hospital" Research-oriented Physician Innovation and Transformation Ability Training Program.

Project Task Book

Project Name: Anticoagulation decision-making and management model based on the "I-Anticoagulation" app in patients with atrial fibrillation: a prospective, multicentre, cluster-randomised controlled study

Project Number: SHDC2022CRS035

Undertaking Unit: Department of Pharmacy, Ren Ji Hospital, Shanghai Jiao Tong University School of Medicine, Shanghai

Project leader: Chi Zhang

Implementation period: Jul. 1, 2022-Sept. 30, 2024

Shanghai Shenkang Hospital Development Center

上海交通大学医学院附属仁济医院
临床科研创新培育基金
计划任务书
（2021）

项目名称： 基于药物基因组与药物代谢组多元标志物的新型口服抗凝药物精准用药研究

项目编号： RJPY-LX-008

项目类别： 连续资助项目

项目负责人： 顾智淳

执行期限： 2021年12月1日 至2024年11月30日

上海交通大学医学院附属仁济医院

2021年 12月8日

Clinical Research Innovation and Cultivation Fund of Ren Ji Hospital

Project Task Book
（2021）

Project Name: **Precision dosing of novel oral anticoagulants based on multiple markers of pharmacogenome and drug metabolome**

Project Number： **RJPY-LX-008**

Type of project： **Continuously funded projects**

Project leader： **Zhi-chun Gu**

Implementation period： **Dec. 1, 2021-Nov. 30, 2024**

Ren Ji Hospital, Shanghai Jiao Tong University School of Medicine

Dec. 8, 2021
