# Supplementary material for: A novel shared decision-making (SDM) tool for anticoagulation management in atrial fibrillation: protocol for a prospective, cluster randomized controlled trial
Source: Trials. 2023 Oct 2;24:623. doi: 10.1186/s13063-023-07667-5 (PMC10544439; doi:10.1186/s13063-023-07667-5)
Supplement: Supplementary file 4 — Additional file 4. Ethical approval document. [file 13063_2023_7667_MOESM4_ESM.pdf]

上海交通大学医学院附属仁济医院伦理委员会批准函

Shanghai Jiaotong University School of Medicine, Renji Hospital Ethics Committee Approval Letter

批件号：KY2022-105-B

一、研究基本信息

项目名称	基于“爱抗凝”小程序的抗凝决策与管理模式在房颤患者中的应用：一项前瞻性、多中心、整群随机对照研究				
项目类型	研究者发起研究	药械名称	口服抗凝药	试验分期/器械类型	不适用
申办单位	上海交通大学医学院附属仁济医院			经费来源	自筹
研究性质	国内多中心			本中心角色	组长单位
组长单位	上海交通大学医学院附属仁济医院			参与单位	花木社区卫生服务中心、金杨社区卫生服务中心、浦兴社区卫生服务中心等 9 家单位
承担科室	药剂科			项目负责人	顾智淳

研究方法		使用生物样本数据	样本来源	样本类型	遗传办公室审批事项
	○实验性研究				
	●前瞻性观察性研究		○既往留存	单击此处输入文字。	○采集 ○利用 ○出境
	○回顾性观察性研究		○计划采集	单击此处输入文字。	○保藏-建立样本库
	○现况性观察性研究		○生物样本库	单击此处输入文字。	○保藏-建立数据库
	○描述性研究	●否	○其他	单击此处输入文字。	○保藏-国际合作

试验用药械是否拟申请注册/申报	否	试验用药械遵循批准适应症范围/使用方法	是

二、审查情况

审查时间	审查方式	应到人数	实到人数	投票人数	利益冲突回避人员
2022 年 06 月 08 日	会议审查	13	13	13	无
2022 年 06 月 22 日	快速审查	不适用	不适用	不适用	无

会议审查项目批件附：《会议签到表》。快速审查项目若需要委员名单请至医院官方网站打印。

三、批准使用的研究文件

●主要研究者及研究团队利益冲突申明信		
●经费来源情况的说明信		
●临床研究方案	版本号：3.0	版本日期：2022.06.16
●知情同意书	版本号：2.0	版本日期：2022.06.16
●招募方式说明	版本号：1.0	版本日期：2022.01.18
●纸质病例报告表	版本号：1.0	版本日期：2022.01.18
●研究者手册	版本号：1.0	版本日期：2022.01.18
●研究团队名单、研究者履历及 GCP 证书、中心列表		
○受试者相关的其他文件：		

四、伦理委员会对研究者及申办者的具体要求

本伦理委员会审查标准依行：卫生部《涉及人的生物医学研究伦理审查办法》(2016)、CFDA《药品临床试验质量管理规范(2003)》、CFDA《药物临床试验伦理审查工作指导原则》（2010）、《医疗器械临床试验质量管理规范》（2016）、《体外诊断试剂临床试验技术指导原则》、《中华人民共和国人类遗传资源管理条例》（2019）、WMA《赫尔辛基宣言》（2013）、CIOMS《人体生物医学研究国际道德指南》（2016）和 ICH-GCP 的伦理原则。该项目审查时，到会人数及有效投票数符合法定要求。经本伦理委员会审查，同意按所批准的临床研究方案、知情同意书、招募材料开展本项研究。

- 研究开始前，请申请人完成临床试验注册。
- 涉及人类遗传资源采集、保藏（建立样本库、建立数据库、国际合作）、利用、出境的研究项目，须在获得人类遗传办公室的批件或备案成功后开展本研究。
- 研究过程中若变更研究者，对临床研究方案、知情同意书、招募材料的任何修改，请申请人提交修正案审查申请。
- 发生严重不良事件，请申请人及时提交严重不良事件报告。
- 请按照伦理委员会规定的年度/跟踪审查频率，申请人在截止日前一个月提交研究进展报告；申办者应当向组长单位伦理委员会提交各中心研究进展的汇总报告；当出现任何可能显著影响试验进行、或增加受试者危险的情况时，请申请人及时向伦理委员会提交书面报告。
- 研究纳入了不符合纳入标准或符合排除标准的受试者，符合终止试验规定而未让受试者退出研究，给予错误治疗或剂量，给予方案禁止的合并用药等没有遵从方案开展研究的情况；或可能对受试者的权益/健康以及研究的科学性造成不良影响等违背 GCP 原则的情况，请申办者及研究者提交违背方案报告。
- 申请人暂停或提前中止/终止临床研究，请及时提交暂停/终止研究报告。
- 重新启动中止/暂停的研究，请在启动前提交重新启动申请。
- 完成临床研究，请申请人提交研究研究总结报告。

**跟踪审查频率**	○ 3个月　○ 6个月　● 12个月　○其他：		
**主任委员**	（签名）	**医学伦理委员会**	（盖章）
**批件生效日期**	2022年6月22日		
**批件失效日期**	2023年6月21日		

伦理委员会联系人：陆麒（医学伦理委员会办公室主任），联系电话：021-68383364，联系地址：上海市浦东新区浦建路 160 号

Shanghai Jiaotong University School of Medicine, Renji Hospital Ethics Committee

Approval No. KY2022-105-B

Ⅰ Information of Research

**Project Name**	Anticoagulation decision-making and management model based on the "I-Anticoagulation" app in patients with atrial fibrillation: a prospective, multicentre, cluster-randomised controlled study				
**Project Type**	Researchers initiate studies	**Medicinal Device Name**	Oral anticoagulants	**Trial Stages/ Medicinal Device Type**	NA
**Applied by**	Shanghai Jiaotong University School of Medicine, Renji Hospital		**Financial Support**	No	
**Research quality**	Multicentre		**This Central Role**	Group leader unit	
**Leader Institute**	Shanghai Jiaotong University School of Medicine, Renji Hospital		**Engaging Institute**	Hua Mu Community Health Service Centre, Jin Yang Community Health Service Centre, Pu Xing Community Health Service Centre and 9 other units	
**Department**	Pharmacy		**Principal Investigator**	Zhi-chun Gu	

**Types of Research**		**Use of biological sample data**	**Sources of Sample**	**Sample Type**	**Genetics Office Approval Matters**
	○ Experimental Research<br>● Prospective observational studies<br>○ Retrospective observational studies<br>○ Current observational studies<br>○ Descriptive research	●No	○Past retention		○ Acquisition<br>○Utilisation ○Exit<br>○ Preservation - Establishment of a sample bank<br>○ Preservation - Establishment of a database<br>○ Preservation - international cooperation
			○Schedule acquisition		
			○ Biobanking		
			○Other		

**Whether the medicinal device intends to apply for registration/declaration**	N/A	**The medicinal device follows the approved indications/method of use**	YES

Ⅱ Review Information

**Review time**	**Review fashion**	**Quorum**	**Number attended**	**Voters**	**Staff of conflict of Interests to avoid**
	Conference review	13	13	13	No
	Rapid review	N/A	N/A	N/A	No

Ⅲ **Approval of Files for Research**		
● Principal investigator information and Statement of conflict of interest of research group		
● Statement of financial support		
●Clinical research plan	Version：3.0	Date of Version：Jun.16,2022
● Informed consent form	Version：2.0	Date of Version：Jun.16,2022
● Statement of recruitment methods	Version：1.0	Date of Version：Jan.18,2022
● Case Report Form	Version：1.0	Date of Version：Jan.18,2022
● Handbook for Researchers	Version：1.0	Date of Version：Jan.18,2022
●List of research group, Curriculum vitae of researchers and GCP		
○Other files of subjects：		

Ⅳ Specific Requirements of Ethics Committee to Researchers and Applicants

The review standards of this ethics committee follow: Ministry of Health "Measures for Ethical Review of Biomedical Research Involving Humans:" (2016), CFDA "Good Clinical Practice for Drugs (2003)", CFDA "Guidelines for Ethical Review of Drug Clinical Trials" (2010), "Quality Management Standards for Clinical Trials of Medical Devices" (2016), "Technical Guidelines for Clinical Trials of In Vitro Diagnostic Reagents", "Regulations of the People's Republic of China on the Administration of Human Genetic Resources" (2019) > WMA Declaration of Helsinki (2013)), the CIOMS International Ethical Guidelines for Biomedical Research in Humans (2016) and the ethical principles of the ICH-GCP. When the project is reviewed, the number of participants and the number of valid votes meet the statutory requirements. After review by the ethics committee, it was agreed to carry out this study according to the approved clinical research protocol, informed consent and recruitment materials.

■ Before the start of the study, the applicant should complete the clinical trial registration.

■ For research projects involving collection, preservation (establishment of sample bank, establishment of database, international cooperation), utilization and export of human genetic resources, this research must be carried out after obtaining the approval or filing of the Office of Human Genetics.

■ If the researcher is changed during the research process, and any modification to the clinical research protocol, informed consent form, and recruitment materials, the applicant is requested to submit an application for amendment review.

■ In the event of serious adverse events, applicants are requested to submit serious adverse event reports in a timely manner.

■ Please submit the research progress report one month before the deadline according to the annual/follow-up review frequency specified by the ethics committee; the sponsor should submit a summary report of the research progress of each center to the ethics committee of the team leader; or increase the risk of subjects, please submit a written report to the ethics committee in a timely manner.

■ Subjects who did not meet the inclusion or exclusion criteria were included in the study, the subjects were not allowed to withdraw from the study in compliance with the regulations for termination of the trial, the wrong treatment or dose was given, the concomitant medication prohibited by the protocol was given, and the study did not follow the protocol ; or may cause adverse effects on the rights/health of subjects and the scientific nature of the research and other situations that violate the principles of GCP, the sponsor and investigator are requested to submit a protocol violation report.

■ If the applicant suspends or prematurely suspends/terminates the clinical study, please submit the suspension/termination report in time.

■ To restart a discontinued/suspended study, please submit a restart request prior to initiation.

■ After completing the clinical research, please submit the research summary report.

Review Frequency	○3 months ○6 months ●12 months ○Other:		
Chairman		Ethics Committee	
Effective date of approval	Jun.22,2022		
Expiration date of approval	Jun.23,2023		
Correspondence of Renji Hospital Ethics Committee: Qi Lu (Chief), Phone: 021-68383364, Address: Shanghai, Pudong district Pujian Road 160			
